# Optimization of the Silver Nanoparticles PEALD Process on the Surface of 1-D Titania Coatings

**DOI:** 10.3390/nano7070193

**Published:** 2017-07-24

**Authors:** Aleksandra Radtke, Tomasz Jędrzejewski, Wiesław Kozak, Beata Sadowska, Marzena Więckowska-Szakiel, Ewa Talik, Maarit Mäkelä, Markku Leskelä, Piotr Piszczek

**Affiliations:** 1 Faculty of Chemistry, Nicolaus Copernicus University in Toruń, Gagarina 7, 87-100 Toruń, Poland; piszczek@chem.umk.pl; 2 Nano-Implant Ltd., Gagarina 5, 87-100 Toruń, Poland; 3 Faculty of Biology and Environmental Protection, Nicolaus Copernicus University in Toruń, Lwowska 1, 87-100 Toruń, Poland; tomaszj@umk.pl (T.J.); wkozak@umk.pl (W.K.); 4 Faculty of Biology and Environmental Protection, University of Lodz, Banacha 12/16, 90-237 Łódź, Poland; beata.sadowska@biol.uni.lodz.pl (B.S.); marzena.wieckowska@biol.uni.lodz.pl (M.W.-S.); 5 A. Chełkowski Institute of Physics, University of Silesia, Uniwersytecka 4, 40-007 Katowice, Poland; ewa.talik@us.edu.pl; 6 Department of Chemistry, University of Helsinki, P.O. Box 55, FI-00014 Helsinki, Finland; maarit.makela@helsinki.fi (M.M.); markku.leskela@helsinki.fi (M.L.)

**Keywords:** plasma enhanced atomic layer deposition, silver nanoparticles, titania nanotubes, titania nanoneedles, bioactivity

## Abstract

Plasma enhanced atomic layer deposition (PEALD) of silver nanoparticles on the surface of 1-D titania coatings, such as nanotubes (TNT) and nanoneedles (TNN), has been carried out. The formation of TNT and TNN layers enriched with dispersed silver particles of strictly defined sizes and the estimation of their bioactivity was the aim of our investigations. The structure and the morphology of produced materials were determined using X-ray photoelectron spectroscopy (XPS) and scanning electron miscroscopy (SEM). Their bioactivity and potential usefulness in the modification of implants surface have been estimated on the basis of the fibroblasts adhesion and proliferation assays, and on the basis of the determination of their antibacterial activity. The cumulative silver release profiles have been checked with the use of inductively coupled plasma-mass spectrometry (ICPMS), in order to exclude potential cytotoxicity of silver decorated systems. Among the studied nanocomposite samples, TNT coatings, prepared at 3, 10, 12 V and enriched with silver nanoparticles produced during 25 cycles of PEALD, revealed suitable biointegration properties and may actively counteract the formation of bacterial biofilm.

## 1. Introduction

The wide use of titanium and its alloys in implantology has contributed to the development of research on the osteointegration and the antimicrobial activity enhancement of modern implants [1,2,3]. The one way to ensure the abovementioned properties is to form the TiO_2_-based nanocomposite coatings on the surface of these implants [4,5,6]. 1-D titania materials of different nanoarchitectures, such as nanotubes (TNT), nanoneedles (TNN), nanowires (TNW), and nanofibers (TNF), have been intensively studied due to their potential applications as biointegrated matrix of formed nanocomposities [7,8,9,10,11,12,13,14,15,16,17]. The high porosity, the high surface to volume ratio, and the morphology (similar to the natural extra-cellular matrix) exhibited by these titania coatings seem to be suitable for this purpose [18,19,20,21,22,23,24,25]. Successful osteointegration is an important clinical goal; however, it should be pointed out that the same important problem is the reduction of bacterial biofilm formation on the surface of the implant. The initial inflammation response is always present regardless of the type of biomaterial used. It may turn into an acute inflammation or even chronic inflammation, for which long-lasting and expensive antibiotic therapy must be used. So it is not surprising that the possibilities of a bacteria-repellent surface modification are being investigated. The previous reports and results of our studies showed that controlled diameter nanotubes displayed significantly changed responses to *S. aureus* and *S. epidermidis* [26,27,28,29]. The tube-size effect is an important factor for antibacterial activity of this type of layers; however, also their structure significantly influences the direction of these changes. According to Puckett et al., the use of larger diameter nanotubes decreased the number of live bacteria as compared to lower diameter ones and pure titanium [30]. However, it should be pointed out that analyzed nanotube coatings were crystalline, in the form of anatase, as they were post-treated after anodization process by annealing. According to results of studies on nanotubes, which were not post-annealed, the best antibacterial properties against *S*. *aureus* were seen for the nanotubes with small diameter but possessing the rutile form [31].

The silver antimicrobial properties have been known for many centuries all around the world. Silver dollars used to be put into bottles with milk in order to keep it fresh, moreover “silvered” water tanks, which were mounted on ships and airplanes were able to render water potable for months [32,33,34,35]. The use of silver as a bactericidal agent decreased when antibiotics were discovered. However, with the discovery of antibiotics, the problem of the emergence of antibiotic-resistant strains appeared. This increasing antibiotic resistance causes the interest in using silver as an antibacterial agent. Silver as antimicrobial agent was analyzed in the form of ions and nanoparticles. The latter ones have been heavily studied because of their biocidal activity. The impact of nanoparticle size on the antibacterial effectiveness was studied by Martinez-Castanon et al. (2008) [36]. Nanoparticles with 7, 29, and 89 nm diameters were synthesized and minimum inhibitory concentration assays (MIC) were performed on *E. coli* and *S. aureus* using the synthesized nanoparticles. The results of the MIC assays showed that smaller nanoparticles inhibited more effectively than larger nanoparticles and that *S. aureus* is more resistant to silver nanoparticles than *E. coli*. In addition to the size, the nanoparticle shape was also analyzed as the parameter, which plays a role in antibacterial activity. Pal et al., (2007) synthesized spherical, rod-shaped, and triangular silver nanoparticles and tested each of them for antimicrobial activity using *E. coli*. It was determined that triangular nanoparticles possessed the most antibacterial properties, spherical ones have almost the same biocidal activity, as triangular ones, whereas the weakest biocidal activity was seen for rod-shaped ones [37]. Barhoum et al., reported on low cost, environmentally friendly and very simple method to control the size, morphology and dispersibility of silver nanocrystals through their synthesis onto the surface of solid nanocrystal support, which should avoid extensive aggregation of silver nanoparticles during the synthesis [38]. The aggregation of silver nanoparticles leads to a loss of surface area and to decrease the antibacterial activity, so it is advantageous to prevent such situation [39].

However, not only benefits of using silver nanoparticles should be taken into account when silver is applied in biomaterials. Potential cytotoxic effect of silver for eucaryotic cells should also be considered. This topic is so large that it would fill the content of several dozen publications, but here the authors would like to emphasize the need to control also these properties of silver. Antimicrobial ability of silver particles is attributed to the strong oxidative activity of nanoparticles surfaces and the release of silver ions to biological environments. Both factors are thought to trigger a series of negative effects on the functions of cells, inducing cytotoxicity, genotoxicity, and even cell death. Both AgNPs and Ag ions can produce oxygen radicals and cause oxidative stress in cells. AgNPs induce stronger oxidative damage to cell membrane and organelles, and oxidative stress caused by silver can trigger inflammatory responses, including the activation of innate immunity. Several factors such as size, shape, surface chemistry, dose, and exposure time, play important roles in mediating cellular responses [40]. One of the method to control the toxicity of silver is to control of its content, especially the content of Ag ions, which are released during the immersion of silver decorated material in aqueous solutions. The release dynamics of Ag should be analyzed to evaluate if the silver release amount is at a safe range. Guo et al., pointed out the maximum Ag concentration released in vitro should be no more than 10 mg/dm^3^ because higher concentration of Ag becomes toxic to human cells [41]. In case of silver concentration oversized the secure range mentioned above, it is safer to use antibiotics as antibacterial agent than to risk the toxic effects of silver [42].

Considering previous reports we have focused on the design and the fabrication of 1-D titania nanoarchitectures decorated by dispersed silver nanoparticles of the strictly defined size and of the biological activity related to the size effect. For this purpose we have chosen the atomic layer deposition (ALD), as a method to enrich titania coatings. ALD relies on cyclewise and alternating exposure to precursor and reactant gases, which interact with the surface of substrate, in self-limiting reactions [43,44,45]. Such nature of surface reactions provides the same amount of deposited material on the entire surface and in this way the excellent film uniformity even on large substrates. Atomic layer deposition allows the control of film thickness at the atomic level, enabled the layer-by-layer film deposition. Simultaneously it is known that silver ALD suffers from poor nucleation and initiates an island growth [46,47]. Therefore many ALD cycles are necessary to obtain a continuous film. We decided to optimize the number of ALD cycles at the adequately low level to obtain dispersed silver nanoparticles rather than continuous film and in this way to control the particle’s nucleation and growth.

In this paper, we discuss the results of our works concerning the deposition of dispersed silver nanoparticles onto 1-D titania nanotube and nanoneedle coatings using plasma enhanced atomic layer deposition (PEALD) technique. The assumption was to obtain a TiO_2_/Ag nanocomposite layer on the Ti6Al4V substrate, in order to: (a) increase biointegration properties of titanium alloys, resulting from titania presence, and (b) give the TiO_2_ layer antimicrobial activity, resulting from the Ag grains existence. The bioactivity and the potential usefulness of produced TiO_2_/Ag coatings in the modification of implants surface have been estimated on the basis of biocompatibility analysis (the fibroblasts adhesion and proliferation studies), and by the determination of their ability to reduce bacterial biofilm formation. In the same time the cumulative silver release profiles have been checked, in order to exclude potentially cytotoxic silver decorated systems.

## 2. Results

### 2.1. The Production of Titania 1-D Coatings (TNT and TNN) and Their Characterization

Titania based coatings were produced on the surface of Ti6Al4V substrates by electrochemical oxidation (TiO_2_ nanotubes (TNT)) or by thermal oxidation (TiO_2_ nanoneedles (TNN)) according to the early reported procedure [48,49,50]. The TNT layers were produced in the range 3–20 V and the time of anodization was 20 min. Analysis of GAXRD (Glancing Angle X-ray Diffraction), Raman spectroscopy, and SEM data proved that obtained coatings are amorphous (as there is lack of signals and bands, which could be assigned to anatase or rutile signals and bands on GAXRD and Raman spectra, adequately; Figures S1 and S2 in Supplementary Materials) and possess nanotubular architecture. They were composed of vertically aligned titania nanotubes, of the length 120–150 nm, which was in the agreement with our earlier results [31,48]. The diameter of the produced nanotubes changed in the range ca. 10–85 nm, depending to the applied anodization potential. Coatings produced between 3 and 8 V (TNT3-TNT8) consisted of densely packed nanotubes, whereas the layers obtained at higher potentials: 10–20 V (TNT10-TNT20) were formed by the separated nanotubes. The thermal oxidation process of Ti6Al4V substrates was carried out under Ar atmosphere at 475 and 500 °C. The oxygen, which was present in argon as impurity (<5 ppm of oxygen), was responsible for the formation of titania thin layer. The morphological studies proved that TiO_2_ coatings produced at 475 °C were composed of dense packed and morphologically well oriented nanowires, with nanoneedle morphology (TNN475) and coatings obtained at 500 °C composed rather of faceted crystals, than needles (TNN500). GAXRD and Raman spectra of titania nanoneedles, which are provided in Supplementary Materials (Figures S3 and S4) proved the rutile form of these coatings.

### 2.2. The Deposition of Silver Particles on the Surface of Titania 1-D Coatings (TNT and TNN) by PEALD and the Characterization of Systems: TNT/Ag and TNN/Ag 

**Figure 1:**
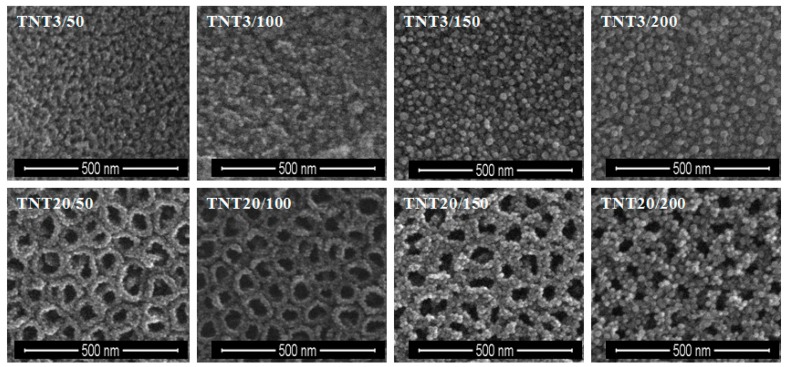
SEM images of TNT/Ag nanocomposite samples consisting of TNT layers prepared at 3 V and 20 V, and enriched with silver grains during 50, 100, 150, 200 ALD cycles.

**Figure 2:**
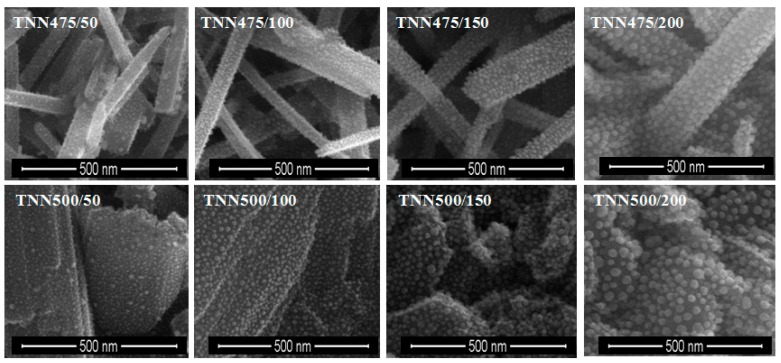
SEM images of TNN/Ag nanocomposite samples consisting of TNN layers prepared at 475 and 500 °C, and enriched with silver grains during 50, 100, 150, 200 ALD cycles.

**Figure 3:**
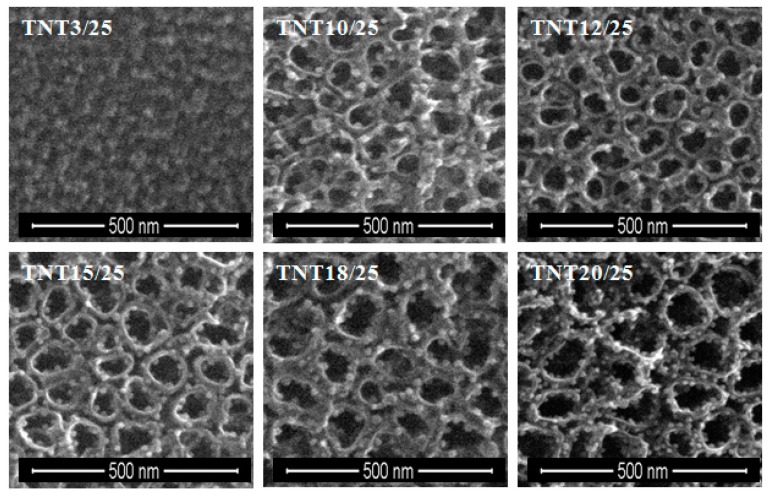
SEM images of TNT/Ag nanocomposite samples consisting of TNT layers prepared at 3, 10, 12, 15, 18, and 20 V, enriched with silver grains during 25 ALD cycles.

Silver particles were deposited on TNT and TNN coatings using Ag(fod)(PEt_3_) as an ALD silver precursor and known procedure [47]. The number of ALD cycles was determined as 50, 100, 150, and 200. SEM images of the obtained TNT/Ag and TNN/Ag samples are presented in Figure 1 and Figure 2. Analysis of SEM images revealed that the use of 50–200 ALD cycles led to the formation of dispersed Ag nanoparticles on the surface of TNN layers (Figure 2). While, the dense packed Ag grains were deposited in the same conditions on the surface of TNT layers (Figure 1). Therefore, for TNT coatings the additional deposition experiments with the use of 25 ALD cycles have been carried (Figure 3). According to the morphological studies, we can state that TNT3-TNT8 samples (diameter of the tube around 10–35 nm) were completely coated by dense packed Ag particles. On the other hand, titania nanotubes of larger tube diameters (TNT10-TNT20) have been coated by dispersed metallic nanoparticles. Considering the potential application of TNT/Ag composites as a biomedical material as it was mentioned in the introduction, only TNT10/25-TNT20/25 samples have been chosen for biological experiments. Moreover, in order to estimate the bioactivity of dense packed Ag grain layers, the TNT3/25 system has been also studied.

**Figure 4:**
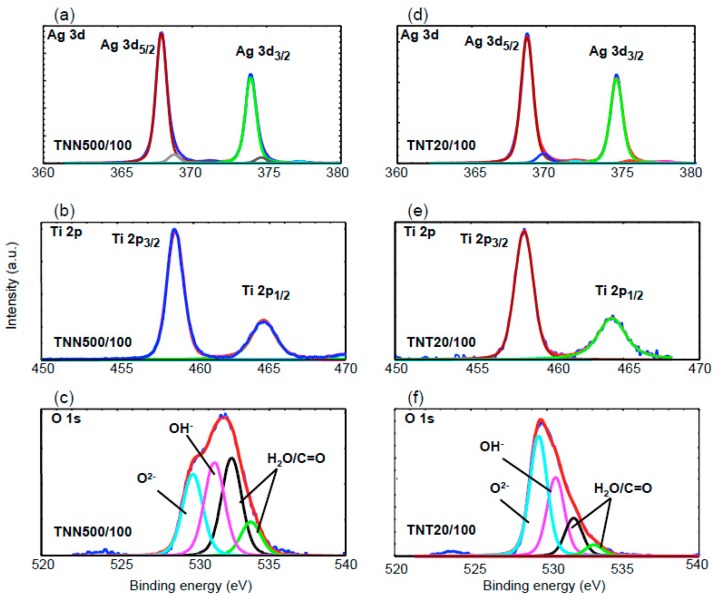
XPS spectra of TNT/Ag and TNN/Ag coatings; (**a**,**d**) Ag 3d, (**b**,**e**) Ti 2p peaks, and (**c**,**f**) O 1s peaks.

X-ray Photoelectron Spectroscopy (XPS) has been used to study the bonding and the status of silver grains deposited on the surface of TNT and TNN layers. Results of these investigations are presented in Table 1 and displayed in Figure 4. In XPS spectra of TNT and TNN samples, the Ti 2p cores split into 2p_3/2_ (458.0–458.7 eV) and 2p_1/2_ (463.9–464.8 eV) peaks, which corresponds to the reported values for Ti^4+^ titanium dioxide [51,52,53,54]. The deconvolution of the O 1s asymmetric peak revealed the presence of four components, which correspond to: (a) the lattice oxygen O^2−^ (529.5–529.9 eV), (b) hydroxide groups (530.5–531.4 eV), and (c) adsorbed water molecules (532.2–532.5 eV) or (d) other oxygen based molecules adsorbed on the TiO_2_ layer surface, (e.g., C=O 533.2–533.7 eV) (Table 1, Figure 4) [55,56,57].

Figure 4 shows XPS spectrum of Ag 3d region for silver nanograins deposited on the surface of TNT (Figure 4a) and TNN (Figure 4d) coatings. The use of the deconvolution methods revealed that Ag 3d peaks consist of one, two or three doublets respectively to the sample type. The first of them consists of peaks at 368.2–368.9 eV and 374.2–374.8 eV, which can be assigned to Ag 3d_5/2_ and Ag 3d_3/2_ respectively. The 6.0 eV separation between the binding energy of these peaks confirms the presence of metallic Ag atoms in deposited grains [54,55,56,57]. In XPS spectra of TNT6/100 and TNN500/100 peaks at 367.8, 373.9 eV and 367.9, 373.9 eV have been found. According to literature reports, these binding energy values are characteristic for interacted Ag^+^ ions with the TiO_2_ surface [58]. Analysis of XPS spectra of further samples showed that the second doublets were found at 369.2–369.9 eV and 375.1–375.6 eV (Table 1, Figure 4d). According to Rodriguez-Gonzalez et al., the 3d Ag peaks at ca. 369.4 and 375.4 eV could suggest the presence of silver particles of different size [59].

### 2.3. Fibroblast Adhesion and Proliferation on TNT/Ag Detected by MTT ((3-(4,5-dimethylthiazole-2-yl)-2,5-diphenyl tetrazolium bromide) Assay

**Figure 5:**
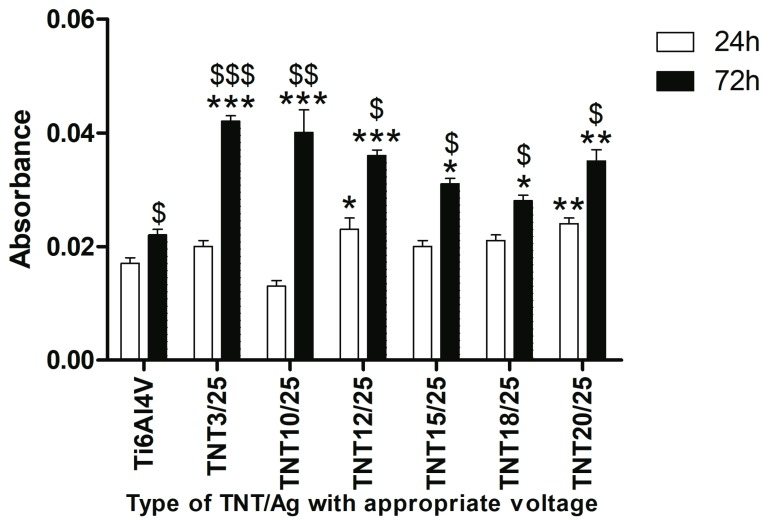
Effect of TiO_2_ nanotubes coatings enriched with silver nanoparticles in PEALD process with 25 cycles on the murine fibroblasts L929 adhesion (after 24 h) and proliferation (after 72 h) detected by MTT assay. The absorbance values are expressed as means ± SEM of three independent experiments. Asterisks indicate significant differences between the fibroblasts incubated with Ti6Al4V alloy references sample (Ti6Al4V) compared to the tested TNT/Ag at appropriated incubation time (* *p* < 0.05, ** *p* < 0.01, *** *p* < 0.001, respectively). $ marks denote significant differences between the cells incubated with TNT/Ag for 24 h compared to 72-h incubation time (^$^ *p* < 0.05, ^$$^ *p* < 0.01, ^$$$^ *p* < 0.001, respectively).

Taking into account results of our earlier investigations of titania nanoneedles (TNN) and their relatively low biointegration activity, we have decided to exclude TNN/Ag from biological research [50]. Bioactivity studies of TNT/Ag nanocomposities have been carried out for systems containing dispersed Ag nanoparticles (TNT10/25-TNT20/25) and for the TNT layer coated by dense packed Ag nanoparticles (TNT3/25). Figure 5 presents the effect of TNT3/25, TNT10/25-TNT20/25 coatings on the L929 murine fibroblasts adhesion (after 24 h) and proliferation (after 72 h) examined using MTT assay. As it can be seen, an increase in the cell adhesion was noticed only in the case of TNT12/25 and TNT20/25 (*p* < 0.05 and *p* < 0.01, respectively) in comparison to the reference sample (Ti6Al4V alloy). In contrast, the results from 72 h incubation time revealed an increase in the level of fibroblasts proliferation for the all tested types of TNT compared to Ti6Al4V alloy, however, this effect was particularly observed in the following samples: TNT3/25, TNT10/25 and TNT12/25 (*p* < 0.001; Figure 5). Our results showed also that with an increase of incubation time, more cells proliferated on the surface of all tested specimens, including Ti6Al4V. Comparing the results obtained for 24 and 72 h incubation, it can be noticed that this phenomenon was particularly observed in the case of TNT3/25 and TNT10/25 (*p* < 0.001 and *p* < 0.01, respectively).

### 2.4. Cell Morphology, Adhesion and Proliferation Observed by Scanning Electron Microscopy

**Figure 6:**
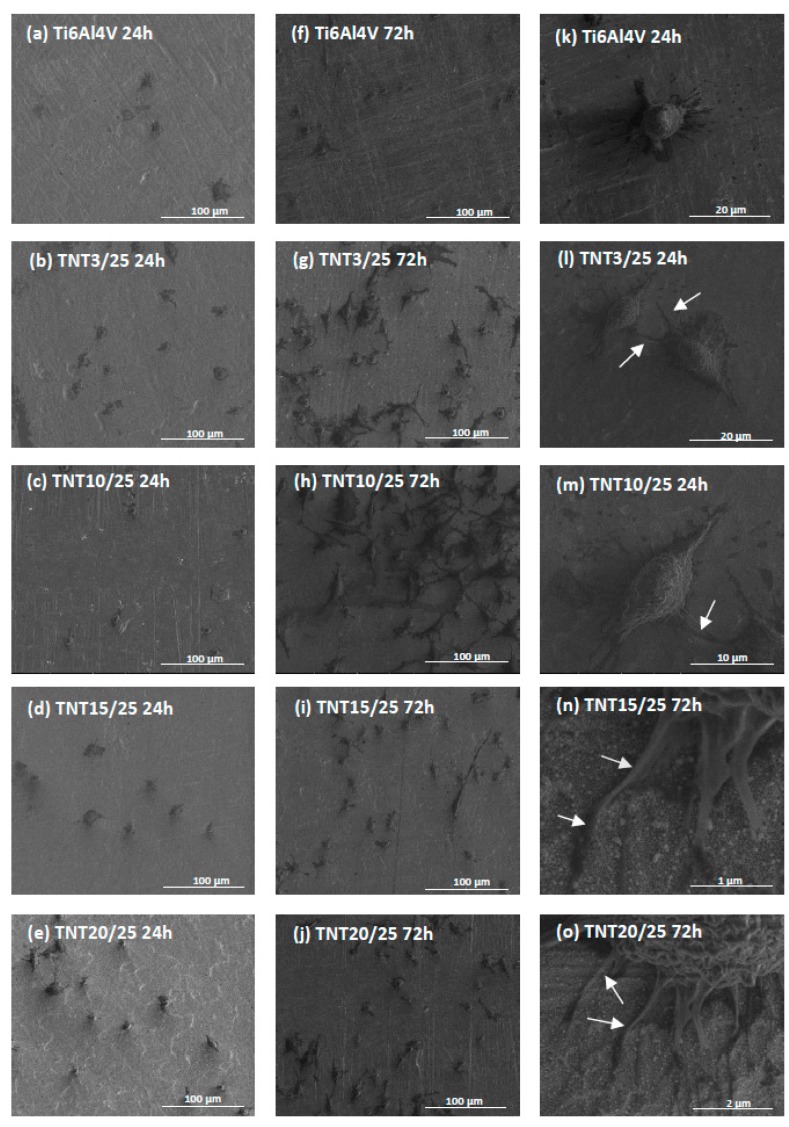
Scanning electron microscopy images showing the adhesion (after 24 h) and proliferation (after 72 h) of L929 murine fibroblasts on the surface of Ti6Al4V alloy and TNT/Ag coatings produces in PEALD process with 25 cycles (TNT3/25, TNT10/25, TNT15/25, TNT20/25) observed after 24 h (**a**–**e**) and 72 h (**f**–**j**) of incubation time, respectively. Figure (**k**) presents rounded shape of the cells incubated with Ti6Al4V reference sample for 24 h in comparison to the more elongated shape of the fibroblasts incubated with TNT3/25 (**l**) and TNT10/25 (**m**) for the same time period. Arrows in the figure indicate the filopodia spread between cells (**l**,**m**) or penetrating deep into the nanolayers (**n**,**o**).

Comparative SEM micrographs of L929 murine fibroblasts cultured on the Ti6Al4V reference sample and TNT3/25, TNT10/25, TNT15/25 and TNT20/25 coatings for 24 h (a–e) and 72 h (f–j) are presented in Figure 6.

Regarding the examination by SEM, the cells cultivated on the TNT/Ag, as well as on Ti6Al4V alloy, effectively attached to the plates surface. It can be seen also that the number of cells attached to the surface of the nanolayers increase together with the duration of incubation time. This phenomenon was more noticeable for tested nanolayers compared to the reference sample. Furthermore, the fibroblasts attached to the Ti6Al4V layers had a more rounded shape after 24 h incubation time (Figure 6k), whereas, those cells cultured on the TNT3/25 and TNT10/25 surface became increasingly more elongated (Figure 6l,m, respectively). Importantly, the cells growing on the TNT alloys formed also filopodia, which attached the fibroblasts to the surface of arrays by penetrating deep into the nanolayers (Figure 6n) or formed them among themselves (Figure 6o).

### 2.5. Antimicrobial Properties Studies on the Base of S. aureus Biofilm Assessment on Titanium Foil

**Figure 7:**
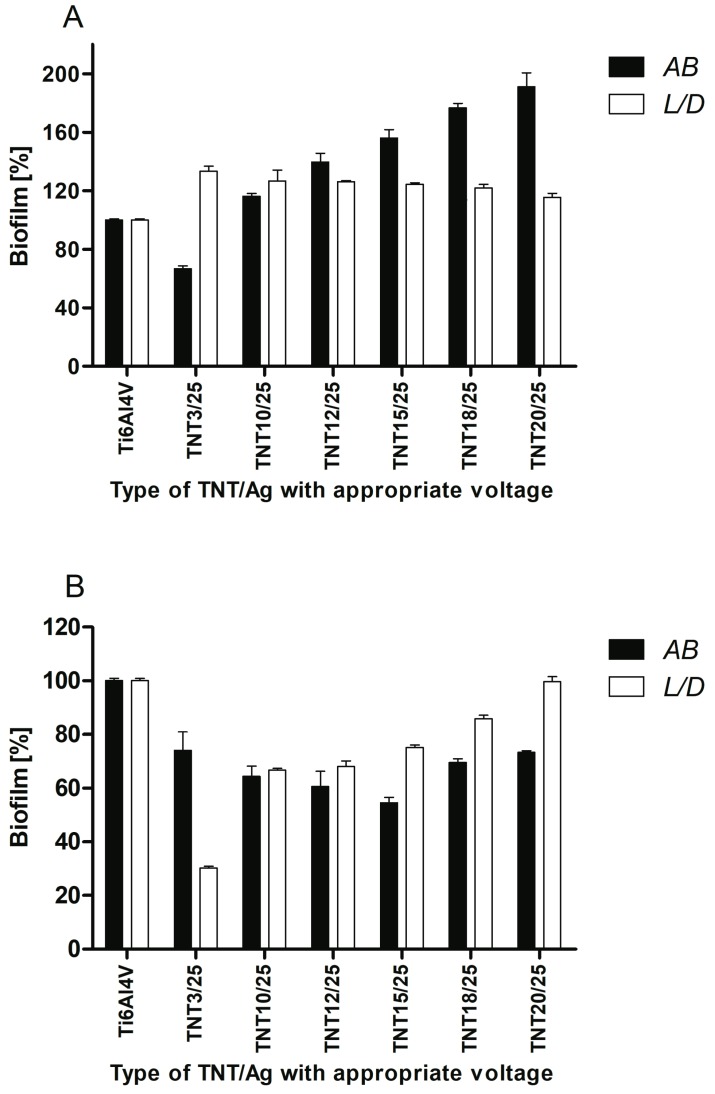
*S. aureus* ATCC 29213 biofilm (24 h old) on the surfaces of titanium nanotubes with dispersed silver particles formed after topical application of bacteria (**A**) or using suspension method (**B**), detected by Alamar Blue assay and Live/Dead BacLight Bacterial Viability kit (alive bacteria). The results are provided as the mean percentage ± standard deviation (SD) of staphylococcal biofilm compared to control biofilm developed on reference sample (Ti6Al4V) considered as 100%. All presented data (except biofilm on TNT20/25 assessed by L/D) were statistically significant (*p* < 0.05).

The samples of titanium nanotubes with dispersed silver particles (TNT/Ag) were exposed for 24 h on staphylococci to assess their ability to inhibit bacterial colonization and biofilm formation. Two different types of microbial contact with biomaterial surface were developed for this study. Topical application of bacteria with limited contact and the suspension method demonstrating complete contact of bacteria with the surface were used to force biofilm formation in series 1 and series 2, respectively. The presence of *S. aureus* ATCC 29213 reference strain biofilm on the surface of TNT/Ag samples was tested using three independent methods: Alamar Blue assay (AB) showing metabolically active cells, Live/Dead BacLight Bacterial Viability kit (L/D) indicating alive and/or dead microorganisms, and the counting of colony forming units (CFU method). The results of AB and L/D methods obtained in series 1 and series 2 are presented in Figure 7A,B, respectively. Considering the topical application of bacteria (series 1) and AB reduction method only TNT3/25 sample was able to reduce significantly biofilm formation of *S. aureus* reference strain (Figure 7A). The average percentage of biofilm inhibition caused by TNT3/25 reached 33.1 ± 1.7% (*p* = 0.0209), in comparison to the control biofilm developed on Ti6Al4V considered as 100%. When microbial biofilm was developed using suspension method (series 2), almost all tested titanium samples caused significant inhibition of biofilm formed by *S. aureus* (Figure 7B). Importantly, such inhibitory activity of titanium samples modified by silver particles was confirmed using both AB and L/D methods. The average percentage of biofilm inhibition achieved the range from 12.31 ± 1.2% (*p* = 0.0202) to 45.61 ± 2.1% (*p* = 0.0209) using AB method and from 14.21 ± 1.3% (*p* = 0.0209) to 69.81 ± 0.7% (*p* = 0.0209) in L/D method in comparison to the control biofilm developed on unmodified titanium alloy considered as 100%. Taking together the results obtained in both series (1 and 2) the sample marked TNT3/25 was the most potent biomaterial able to limit staphylococcal biofilm formation independently from the method of *S. aureus* application. The average percentage of *S. aureus* biofilm inhibition in both experimental series reached 29.51 ± 5.2% for AB method and 34.91 ± 49.4% for L/D.

**Figure 8:**
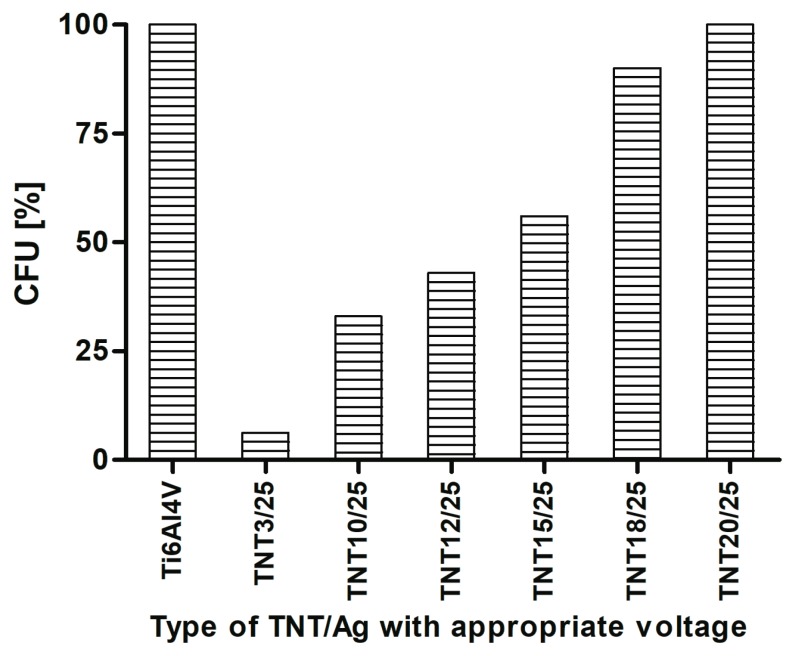
*S. aureus* ATCC 29213 biofilm formation on the surfaces of Ti6Al4V alloy foil modified by TiO_2_ nanotubes (TNT) tested by CFU method. The results are presented as mean percentage ± standard deviation (S.D.) of *S. aureus* CFU reclaimed after 24 h from Ti6Al4V alloy biomaterials modified by TiO_2_ nanotubes with dispersed Ag nanoparticles (TNT/Ag), in comparison to bacterial CFU recovered from control (unmodified) biomaterial (Ti6Al4V) considered as 100%.

Strong anti-biofilm activity of selected TNT/Ag samples in series 2 was also confirmed using CFU method. The most prominent biofilm inhibition was caused by TNT3/25 (93.7%), TNT10/25 (67.0%), TNT12/25 (57.0%), and TNT15/25 (44.0%) (Figure 8).

### 2.6. Silver Ions Releasing from TNT/Ag Nanocomposites

**Figure 9:**
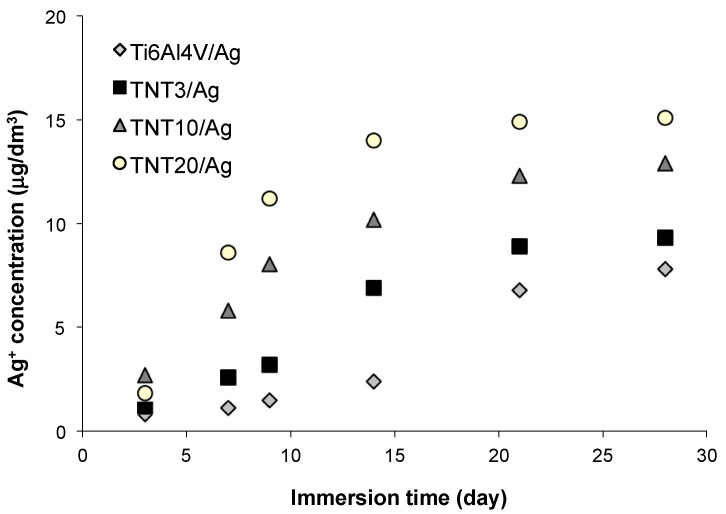
The cumulative silver release profile obtained on the basis of ICPMS data.

Ti alloy/Ag (reference sample) and TNT/Ag nanocomposite coatings were immersed in phosphate buffered saline (PBS) solution at 37 °C (human body temperature) for 1, 3, and 4 weeks in order to study the silver ions releasing (Figure 9). Silver ions release processes are presented on Figure 9. The obtained results showed an inhibition of silver releasing in PBS solution for all studied samples after 3 weeks. The level of releasing is clearly depended to the size of titania nanotubes and it is the highest for the TNT20/Ag, so for the sample with the biggest titania nanotube diameter. In this case, the silver releasing reached after 4 weeks the level of 15 μg/dm^3^ which is still significantly lower than the value, which is toxic to human cells, 10 mg/dm^3^ [41].

## 3. Discussion

**Figure 10:**
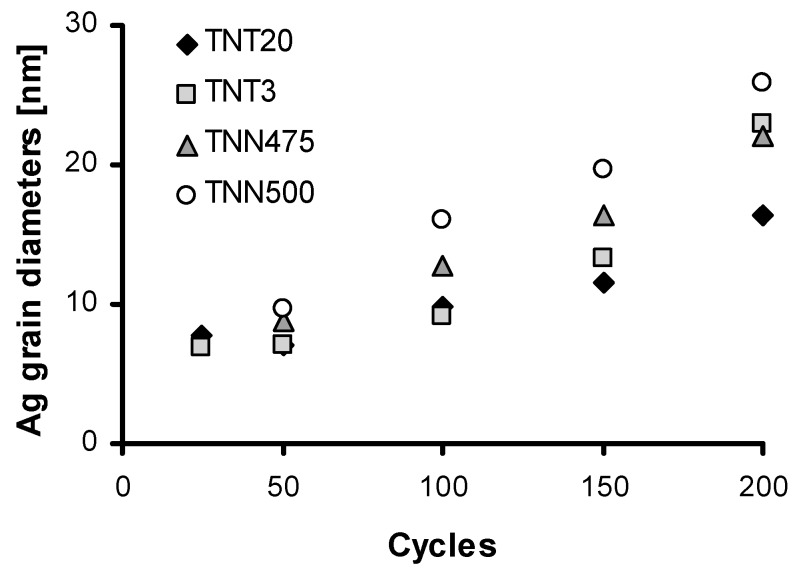
The dependency between the number of cycles and silver grain diameters.

Analysis of SEM images let to evaluate the changes of average diameter values of silver particles deposited during PEALD processes with the use of 25 to 200 cycles (Figure 1 and Figure 2). Generally, it should be noticed that the size of silver nanoparticles depends on the number of cycles and the substrate type (TNT, TNN), which is illustrated in Figure 10. The ALD cycles number increase leads to the increase of Ag grains diameter regardless of the substrate type, which is associated with the continued nucleation and nanoparticles coalescence [60,61]. Morphology studies revealed the noticeable influence of the substrate type on the diameter of the deposited Ag grains. For 50 cycles deposition process, the average diameter of silver nanoparticles was equal to 6.8–7.2 and 8.8–9.6 nm for TNT and TNN, respectively, so the differences were not so significant. The increase of the cycle number up to 100 caused the increase of Ag grain diameters, in TNN475/100 and TNN500/100 systems (Ag grain diameters were equal to 12.7 and 16.1 nm, respectively). In the case of TNT substrates, the diameters of metallic grains were almost identical independently on the tube size (they were estimated as ca. 9.2–9.9 nm for TNT3/100 and TNT20/100, respectively). In the deposition processes with the use of 200 cycles Ag grains of diameters 22.9 nm and 16.3 nm have been received for TNT3/200) and TNT20/200 respectively (Figure 10). Obtained results point out a clear impact of the titania tube size on the diameter of the deposited silver particles. This dependency is especially visible for smaller nanotubes.

From the biological point of view, the dispersion of Ag particles on the TNT and TNN coatings is important. According to images presented in Figure 2, the silver depositions on the surface of TiO_2_ nanoneedles lead to the formation of dispersed silver nanograins. The silver ALD on the surface of titania nanotubes coatings is more complicated process and strictly depends on the tube diameter. SEM studies of TNT coatings produced in the potential range 3–20 V showed that layers produced between 3 and 10 V consist of dense packed amorphic TiO_2_ nanotubes, which diameters vary from ca. 10–15 nm (3 V) up to ca. 35–40 nm (8 V). TNT10-TNT20 coatings consist of separated tubes of diameters ca. 38–43 nm (10 V) and ca. 77–84 nm (20 V). Small tubes diameter of TNT3-TNT8 and their dense packing caused that on their surface the homogeneous layer of silver dense packed grains have been deposited after 150 and 200 ALD cycles (TNT layers are completely covered by Ag nanoparticles, so the tubes topography is not visible, Figure 1). The reduction of the cycles number up to 50 and 100, led to the deposition of densely packed Ag nanoparticles on the top edges of TiO_2_ nanotubes. Increase of tubes diameter (TNT10-TNT20) and their separation caused that Ag particles have been deposited both on the top of tubes and inside of them. However, independently to the number of cycles, Ag dispersed grains were not obtained. In order to receive TNT/Ag composite which consists of dispersed Ag nanograins, the number of ALD cycles has been decreased to 25 (Figure 3). It caused that on the tubes top edges and surfaces of their walls, dispersed nanograins of the diameter 7.8–9.2 nm were formed. XPS studies proved that most of produced TNT/Ag coatings and TNN475/100 contain metallic silver grains. The exceptions are TNT6/100 and TNN500/100 for which the presence of silver ions have been confirmed (14.0% and 94.9%, respectively). According to earlier reports, peaks ca. 367 and 374 eV may imply that silver particles are anchored on surface of TNT and TNN by Ag–O–Ti bonds [54,55,56,57,58,59].

Cellular morphology is a strong indicator of cell activation, communication and cell–biomaterial integration. Thus, scanning electron microscopy (SEM) imaging has been utilized to evaluate cell morphology in order to better understand the biocompatibility of the tested biomaterials. Murine fibroblasts cultivated on the TNT coatings enriched with silver nanoparticles in PEALD process with 25 cycles effectively attached to the plate’s surface and the number of cells attached to the surface of the nanolayers increase together with the duration of the incubation time (Figure 6). The trend shown in the SEM analysis is similar to that demonstrated in MTT assay (Figure 5). The results from the cell viability test revealed that all biological studied nanocomposite coatings enhanced L929 fibroblasts proliferation observed after 72 h compared to Ti6Al4V alloys. Furthermore, as can be seen in Figure 6g–j,l–o cells growing on TNT3/25, TNT10/25, TNT15/25 and TNT20/25 surface form filopodia that spreading between fibroblasts and penetrating deep into the nanolayers. Filopodia act as a partial regulator of cell adhesion, proliferation and cell-cell interactions [62] as well as they have been shown to “sense” biomaterial surface topographies [63]. It is well known that a typical proliferation process involves extension and adhesion of the leading cell edges and cell division. Moreover, the interaction of filopodia with nanotubes may increase their capability to extend and adhere to leading cell edges, subsequently promoting cell communication and proliferation [64]. These findings all together are important since fibroblasts are considered to be the most common cells in connective tissue, one of the main components of peri-implant soft tissue, which is the key to the formation of the peri-implant mucosal seal and which help to prevent epithelial ingrowth [65]. Our results from MTT assay and SEM analysis seem to demonstrate the biocompatible properties of the studied TNT/Ag layers.

The TNT with a smaller diameter (e.g., TNT3: 10–15 nm, TNT10: 38–43 nm) induced a stronger cell proliferation compared to the nanolayers with a larger diameter (e.g., TNT20: 77–84 nm). Importantly, cell growing was tested on the nanotubes that was enriched with silver nanoparticles in PEALD process with the same 25 cycles. Moreover, as we described above (Figure 10), the average diameter of silver grains of these samples (e.g., TNT3/25 and TNT20/25) was almost identical. Based on these results, we presume that the diameter of nanotubes was determining factor for the growth of cells. Our observations were confirmed by Lan et al., who demonstrated that Ag-decorated TiO_2_ nanotubes exhibited a monotonically increasing trend in MRC-5 human fibroblasts cell line proliferation with decreasing nanotube diameter, indicating that the fibroblast cells showed an obvious diameter-dependent behavior on Ag-decorated nanotubes [66]. It has been reported that a 15–25 nm spacing facilitates clustering of integrin resulting in optimal integrin activation, which is essential for cell adhesion and growing, and hence the smaller diameter nanotubes, even those decorated with Ag nanoparticles, had more focal points for the fibroblast cells to get attached and thus aid in cell adhesion and proliferation [67]. Moreover, according to the morphological studies (Figure 3), we have shown that a smaller diameter of the tube caused, that the samples have been completely coated by dense packed silver particles, whereas titania nanotubes with a larger tube diameters have been coated by dispersed metallic nanoparticles. We suppose the decoration of silver nanoparticles modified the surface topography of the nanotubes with a smaller diameter causing more irregular topography on a nanometric scale. In consequence, this topography may provide more suitable nanometric sites for integrin clustering, thus resulting in enhanced cell proliferation.

The efficiency of Ag action results from many targets in microbial cells, including cell wall synthesis, membrane transport, electron transport in respiratory chain, protein function, as well as DNA transcription and translation [67]. Chronic, difficult to treat infections, usually complicated by microbial biofilm formation, such as biomaterial-associated infections (accompanying inserted or implanted medical devices, including intravascular catheters, prosthetic valves, orthopedic and neurosurgical implants, vascular grafts, etc.) or wound infections (e.g., surgical site infections, diabetic foot, venous leg ulceration) force the need to develop a new treatments alternative to classic antibiotics [68,69]. Therefore, recently antimicrobial properties of silver (particularly silver nanoparticles—AgNPs) have attracted much attention again. Lan et al., demonstrated that Ag-decorated TiO_2_ nanotubes could greatly inhibit the growth of *S. aureus* in suspension after 4 h of co-incubation [67]. According to Wan et al., silver ions implanted into the surface of stainless steel, pure titanium and Ti-Al-Nb titanium alloy samples were responsible for antibacterial properties of those biomaterials tested against *S. aureus* [70]. Barhoum et al., reported antibacterial activity against *Escherichia coli* of tiny Ag nanocrystals synthesized via the hot injection approach [38]. In our study, anti-biofilm potential of all TNT surfaces covered by silver particles was generally proved when suspension method to induce microbial biofilm formation was used. The discrepancies observed between topical application of bacteria and suspension method (Figure 7A,B, respectively) were significant since in the first series a modified TNT samples (except TNT3/25) did not reduce *S. aureus* biofilm formation. Many factors can influence such differences, including the size of bacteria-biomaterial contact area, realized type of microbial growth, the possibility of active biomaterial components release in suspension method, as well as the nanostructure of biomaterials tested. Staphylococci applied topically on titanium samples had limited contact with silver particles, which can restrict their antimicrobial activity. The most commonly considered mechanisms of AgNPs action contain: cell wall and membrane permeability leading to the damage/death of microbial cell, induction the release of reactive oxygen species (ROS) and bactericidal free radicals forming, modification of respiratory activity and intracellular ATP level, and inhibition of bacterial DNA replication [38,71]. All of them require direct physically contact of silver (preferably in the form of ions) with bacterial cell as well as also the penetration, and perhaps because of it the viability (L/D method) of *S. aureus* applied topically on TNT was no observed. Moreover, we can speculate that space availability can determine the type of bacterial growth. Bacteria deposited on the surface will preferentially create a biofilm. These in suspension surrounding the surface can grow as planktonic form or attach to the surface to develop a biofilm. If the surface possesses antimicrobial activity, as TNT Ag-decorated samples tested, bacteria can choose free style of life and limit a sessile population. Using the suspension method of biofilm formation not only the broader contact of bacteria with tested titanium samples but the release of some active biomaterial components to staphylococcal suspension can be also expected. The more that Lan et al. [62] showed Ag ion release from the Ag-decorated nanotubes into PBS solution during the incubation time. Such conditions in our research could have allowed TNT samples tested to affect both metabolic activity and viability of *S. aureus*. Independently from the method of bacterial application the general trend of the worse microbial biofilm suppression with the higher the anodization voltage was shown. Similar observation was also made in our previous study pointing at titanium foil anodized at a potential 4 V as the most active biomaterial against staphylococcal biofilm formation. It has been concluded that the nanostructure of titanium surfaces *a* is very important factor affecting microbial colonization, since under potential below 5 V typical amorphous TiO_2_ coatings and nanotubes with diameter about 20–30 nm were developed, while at higher voltage crystalline grains and the greater diameters of nanotubes were obtained [48]. Summarizing, since we suggest suspension method is better reflecting in vivo conditions because of unlimited contact the microorganisms with biomaterials placed in tissues, the results obtained in present study seem to be advantageous in the context of possible use nanostructural Ag-modified titanium as a biomaterial for medical implants.

## 4. Materials and Methods

### 4.1. Ti6Al4V Substrates

Titanium alloy foils (Ti6Al4V, grade 5) were cut into 10 mm × 70 mm and 10 mm × 10 mm pieces. Obtained substrate samples were ultrasonically cleaned sequentially in acetone (15 min), 80% ethanol (5 min), and deionised water (15 min). The substrates were dried in an Argon stream at room temperature. The surface of the substrates which were prepared for the electrochemical oxidation processes were chemically etched in a 1:4:5 mixture of HF:HNO_3_:H_2_O for 30 s, cleaned with deionised water, and dried in Argon stream. The surface of the substrates used in the thermal oxidation process was activated by its immersion in a 1:1 mixture of 36.5% HCl and water at 80 °C for 30 min. After the activation, the samples were cleaned with deionised water and dried in Argon stream.

### 4.2. Electrochemical Oxidation of Ti6Al4V

The electrochemical oxidation of Ti6Al4V was carried out at room temperature using 0.3 wt % aqueous HF solution as electrolyte according to earlier reports [31,48]. The anodization voltage (*U*) was varied from 3 V up to 20 V and the anodization time *t* = 20 min. In order to purify the produced coatings, they were washed with distilled water with the addition of Al_2_O_3_ powder (averaged particle size = 50 nm) in an ultrasonic bath for 1 min, and then dried in Ar stream. Samples obtained at mentioned conditions were denoted as: TNT3-TNT20.

### 4.3. Thermal Oxidation of Ti6Al4V

The thermal oxidation of Ti6Al4V was conducted according known procedure in a vacuum tube furnace at temperatures between 475 and 500 °C under a pressure of 1–1.5 hPa for oxidation time *t* = 2 h [49,50]. The oxidation process was carried out in the presence of argon (Argon Pre-Pure, purity: 99.998%, impurity: oxygen < 5 ppm, moisture < 5 ppm, total hydrocarbons < 2 ppm) using the following flow rate 30 cm^3^/min. Samples obtained at mentioned conditions were denoted as: TNN475 and TNN500.

### 4.4. Characterization of Titania Coatings

The structure of the produced titania based nanomaterials was characterized using glancing angle X-ray diffraction (PANalytical X’Pert Pro MPD X-ray diffractometer using Cu-Kα radiation, PANalytical B.V., Almelo, The Netherlands) the incidence angle was equal to 1 deg), Raman spectroscopy (RamanMicro 200, Perkin Elmer, Waltham, MA, USA). The morphology of the produced coatings was studied using Quanta field-emission gun scanning electron microscopy (SEM; Quanta 3D FEG; Carl Zeiss, Göttingen, Germany).

### 4.5. PEALD of Silver Nanoparticles

Plasma enhanced atomic layer deposition method was chosen to carry out the enrichment process of 1D-titania nanotubes (TNT) and nanoneedles (TNN). [Ag(fod)(PEt_3_)], where fod–2,2-dimethyl-6,6,7,7,8,8,8-heptafluorooctane-3,5-dionato ligand, has been used as silver precursor and depositions were made on PEALD reactor–Beneq TFS 200, with a remote plasma configuration, according known procedure [47]. Plasma activated hydrogen was used as the reducing agent. Hydrogen gas (99.999%, AGA) was mixed with argon (99.999%, AGA) which was also the carrier gas. Hydrogen concentration was about 6.5%. Plasma, which power was 100 W, was generated with capacitive coupling with a 13.56 MHz RF (radio frequency) power source. The hydrogen flow was not pulsed because no reaction between the precursor and molecular hydrogen was noticed at the applied growth temperatures. Gases were purified on-site before mixing with Aeronex GateKeeper and Entergris GateKeeper purifiers. The precursor, Ag(fod)(PEt_3_), was loaded onto an open boat and vaporized at 106 °C. The precursor was pulsed into the reaction chamber with inert gas valving. The substrates were TNT on Ti6Al4V (5 mm × 5 mm) and TNN (10 mm × 10 mm), loaded at the same time for each growth experiment. The metal precursor pulse length was varied between 1 and 4 s and the hydrogen plasma pulse between 3 and 5 s. Typically, the purge length after the metal precursor was 5 s and after the plasma exposure 3 s. The used growth temperatures were 120 °C. The only parameter, which was changed during the deposition series, was the number of cycles: 25, 50, 100, 150, and 200.

### 4.6. Characterization of TiO_2_/Ag Coatings

The structure of the produced titania based nanocomposite materials (TNT/Ag and TNN/Ag) was characterized by X-ray photoelectron spectroscopy (XPS). XPS spectra of investigated samples were obtained with the use of PHI 5700/660 ESCA spectrometer (Physical Electronics, Lake Drive East Chanhassen, MN, USA), with monochromatized Al Kα radiation (1486.6 eV) at room temperature. The morphology of produced coatings was studied using Quanta field-emission gun scanning electron microscopy (SEM; Quanta 3D FEG; Carl Zeiss, Göttingen, Germany).

### 4.7. Cell Adhesion and Proliferation Assay on TNT/Ag

Murine fibroblasts cell line L929 (American Type Culture Collection) culture conditions were the same as we described previously [48,49]. The effect of TiO_2_ nanotube coatings enriched with silver nanoparticles in PEALD process with 25 cycles (TNT) on the L929 cells adhesion (after 24 h) and proliferation (after 72 h and 5 days) was assessed using the MTT (3-(4,5-dimethylthiazole-2-yl)-2,5-diphenyl tetrazolium bromide; Sigma Aldrich; Darmstadt, Germany) assay [43,44]. Scanning electron microscopy (SEM; Quanta 3D FEG; Carl Zeiss, Göttingen, Germany) analyses were performed to study the morphology changes of L929 cells grown on the surface of TNT using the same method as in [48,49].

### 4.8. Statistical Analysis

Statistical significance was determined using one-factor analysis of variance (ANOVA). As a post hoc test the Tukey test was used. The level of significance was set at *p* < 0.05.

### 4.9. Bacterial Strains and Biofilm Formation on Titanium Biomaterials

*Staphylococcus aureus* ATCC 29213 reference strain (MSSA, methicillin-susceptible *S. aureus*) was grown for 24 h at 37 °C on Mueller-Hinton Agar—MHA (BTL, Warszawa, Poland) and next 18 h on Tryptic Soy Broth—TSB (BTL, Warszawa, Poland) containing 0.25% glucose (TSB/Glu). Microbial suspension at the optical density OD_535_ = 0.9 (nephelometer type Densilameter II, Pliva-LACHEMA Diagnostika, Brno, Czech Republic) were prepared. 40 μL of bacterial suspension was topically applied on titanium samples (series 1) or biomaterial samples were added to 1 mL of bacterial suspension (series 2) into the wells of a 24-well tissue culture polystyrene plates (Nunc, Roskilde, Denmark). The biomaterials were incubated with bacteria for 24 h at 37 °C in stable conditions to form microbial biofilm. Staphylococcal culture alone (without biomaterial) and TSB/Glu only were used as a positive and negative control, respectively. Two independent experiments with four replicates in each were performed.

### 4.10. The Assessment of S. aureus Biofilm on Titanium Foil

To evaluate biofilm formation, Alamar Blue (AB; Invitrogen, Thermo Fisher Scientific, Eugene, OA, USA) staining, LIVE/DEAD BacLight Bacterial Viability kit (L/D; BacLight Bacterial Viability kit (L/D; Invitrogen, Thermo Fisher Scientific, Eugene, OA, USA) and CFU method were used. Bacteria weakly bounded with the surface of biomaterials were gently removed and the pieces of titanium foil were vortexed (3 min) in TSB to reclaim the cells forming biofilm. Obtained *S. aureus* suspensions or medium (negative control) were added (100 μL) to the wells of 96-well tissue culture polystyrene microplates (Nunclon Surface, Nunc, Roskilde, Denmark) in quadruplicate to perform AB and L/D test or diluted from 10^−1^ to 10^−5^ in phosphate buffered saline (PBS; IBSS Biomed, Kraków, Poland) and cultured to count CFU (colony forming units) as was previously described [48]. The results were presented as a percentage of metabolically active bacteria (AB method)/alive bacteria (L/D and CFU methods) in biofilm formed on modified titanium foils tested in comparison to microbial biofilm on control unmodified biomaterial, considered as 100%.

### 4.11. Statistical Analysis

The nonparametric Kruskal–Wallis one-way ANOVA was used to compare differences among the samples from different populations. *p* ≤ 0.05 was considered significant.

### 4.12. Silver Ions Releasing

In order to study the silver ions releasing, Ti6Al4V/Ag (reference sample) and TNT/Ag nanocomposite coatings were immersed in phosphate buffered saline (PBS) solution at 37 °C (human body temperature) for 1, 3, and 4 weeks. The silver concentration in the elutes was measured by means of an inductively coupled plasma mass spectrometry device (ICP MS Agilent Technologies 7700X, Tokyo, Japan), calibrated with five dilutions (1.00, 2.50, 5.00, 7.50, 10.00 μg/dm^3^) of the Multi-Element Aqueous CRM Enviromental Calibration Standard A (VHG Labs., Manchester, NH, USA) with an original Ag concentration of 10.00 ± 0.05 μg/dm^3^. Qualitative and quantitative analysis of the concentration of Ag was performed using the software MassHunter Workstation Software for ICP-MS Version A.01.02 G7201A Build 291.22 Patch 5.

## 5. Conclusions

The anodization of Ti6Al4V alloy foil and further ALD of silver nanoparticles allows the formation of nanocomposite layers TNT/Ag, which revealed promising biointegration properties and antibacterial activity. The use of small amount of cycles in ALD method (in our studies—25 cycles) enabled the formation of dispersed metallic Ag particles on the surface of TNT layers. The particular properties of the TNT3/25 show that this coating is promising due to its suitable cell adhesion and proliferation and significant biofilm inhibition properties. Obtained results indicate that also bioactivity of TNT10/25 and TNT12/25 coatings may be suitable for their application in the constriction of the new generation implants, which actively participate in the processes of osteointegration and counteract the formation of bacterial biofilm. All obtained samples were characterized by the low level of silver ions releasing, so they can be treated as non-toxic material for human cells.

## Figures and Tables

**Table 1:** XPS data of TNT/Ag coatings.

	**Ti^4+^**		**O^2−^**	**OH^−^**	**H_2_O**	**C=O**
**TiO_2_ Samples**	**Ti(2p_3/2_) (eV)**	**Ti(2p_1/2_) (eV)**	**O(1s) (eV)**	**O(1s) (eV)**	**O(1s) (eV)**	**O(1s) (eV)**
**TNT3/**50	458.7	464.4	529.9 (47%)	531.0 (32%)	532.2 (17%)	533.5 (4%)
**TNT3/**100	458.5	464.2	529.9 (32%)	531.0 (36%)	532.0 (24%)	533.2 (8%)
**TNT6/**50	458.7	464.4	529.9 (43%)	531.1 (21%)	532.2 (29%)	533.7 (7%)
**TNT6/**100	458.5	464.2	530.0 (45%)	531.1 (31%)	532.3 (16%)	533.4 (7%)
**TNT15/**50	458.2	463.9	529.9 (66%)	531.2 (20%)	532.2 (10%)	533.3 (4%)
**TNT15/**100	458.5	464.2	529.9 (51%)	531.0 (22%)	532.1 (21%)	533.4 (6%)
**TNN500/100**	458.4	464.2	529.9 (27%)	531.4 (30%)	532.5 (32%)	533.8 (11%)
**TNN475/100**	458.0	464.8	529.5 (48%)	530.5 (32%)	532.5 (15%)	533.2 (3%)
	**Ag**		**Ag^+^**		**Ag**	
**TiO_2_ Samples**	**Ag(3d_5/2_) (eV)**	**Ag(3d_3/2_) (eV)**	**Ag(3d_5/2_) (eV)**	**Ag(3d_3/2_) (eV)**	**Ag(3d_5/2_) (eV)**	**Ag(3d_3/2_) (eV)**
**TNT3/**50	368.7 (96%)	374.6 (96%)	-	-	369.8 (4%)	375.4 (4%)
**TNT3/**100	368.4 (96%)	374.4 (96%)	-	-	369.4 (4%)	375.2 (4%)
**TNT6/**50	368.8 (100%)	374.8 (100%)	-	-	-	-
**TNT6/**100	368.5 (82%)	374.5 (82%)	367.8 (14%)	373.9 (14%)	369.4 (4%)	375.3 (4%)
**TNT15/**50	368.2 (97%)	374.2 (97%)	-	-	369.2 (3%)	375.1 (3%)
**TNT15/**100	368.4 (94%)	374.4 (94%)	-	-	369.3 (6%)	375.1 (6%)
**TNN500/100**	368.9 (6%)	374.6 (6%)	367.9 (94%)	373.9 (94%)	-	-
**TNN475/100**	368.8 (97%)	374.8 (97%)	-	-	369.8 (3%)	375.6 (3%)

## Supplementary Materials

The following are available online at http://www.mdpi.com/2079-4991/7/7/193/s1, Figure S1: GAXRD spectrum of TNT20, Figure S2: Raman spectrum of TNT20, Figure S3: GAXRD spectrum of TNN475, Figure S3: Raman spectrum of TNN475.
