# Supplementary material for: Optimization of the Silver Nanoparticles PEALD Process on the Surface of 1-D Titania Coatings
Source: Nanomaterials (Basel). 2017 Jul 24;7(7):193. doi: 10.3390/nano7070193 (PMC5535259; doi:10.3390/nano7070193)
Supplement: Supplementary file 1 [file nanomaterials-07-00193-s001.pdf]

Supplementary material: Figure S1 – Figure S4

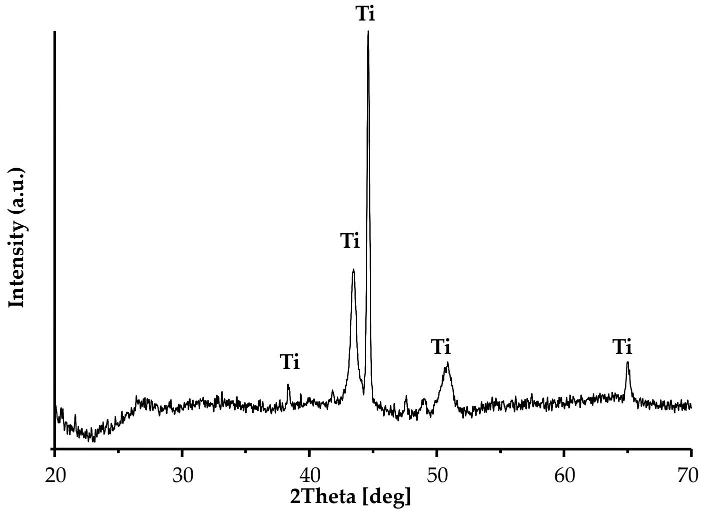

Figure S1. GAXRD spectrum of TNT20

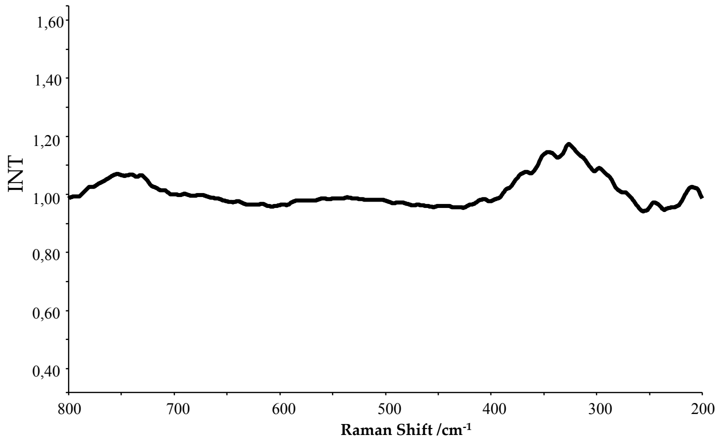

Figure S2. Raman spectrum of TNT20

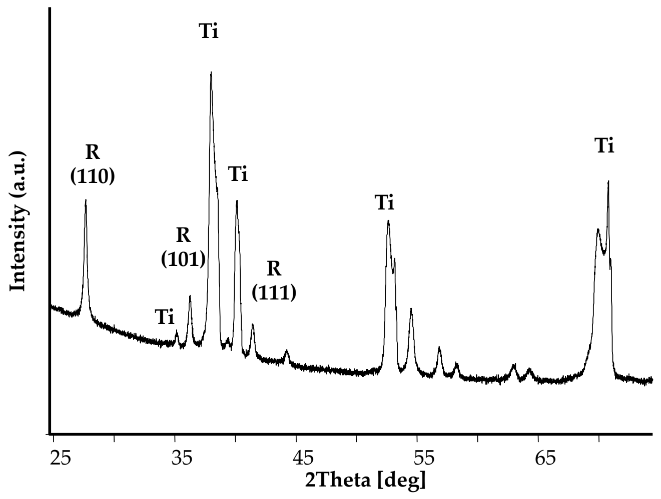

Figure S3. GAXRD spectrum of TNN475

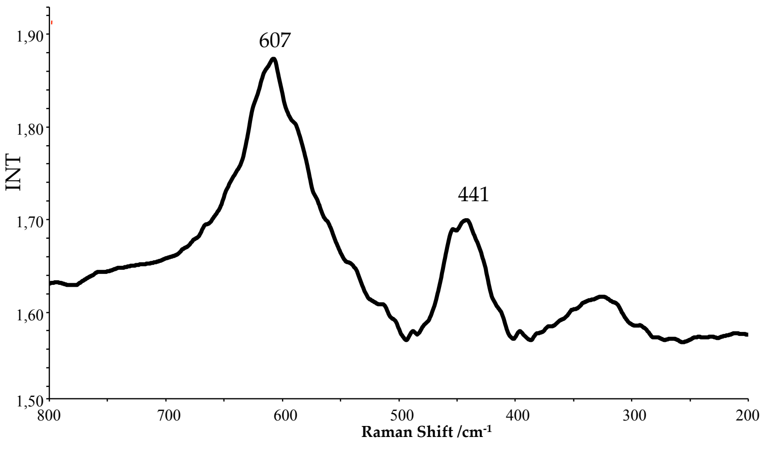

Figure S4. Raman spectrum of TNN475
